# Emergency postpartum hysterectomy as a consequence of cervical varix during pregnancy; a case report and literature review

**DOI:** 10.1016/j.ijscr.2023.108425

**Published:** 2023-06-17

**Authors:** Nafisseh Saedi, Marjan Ghaemi, Mona Moghadam, Mohammad Haddadi, Zeinab Hashemi, Sedigheh Hantoushzadeh

**Affiliations:** Vali-E-Asr Reproductive Health Research Center, Family Health Research Institute, Tehran University of Medical Sciences, Tehran, Iran

**Keywords:** Cervical varix, Vaginal bleeding, Transvaginal ultrasound, Color Doppler, Maternal morbidity, Case-report

## Abstract

**Introduction and importance:**

Vaginal bleeding might accrue during pregnancy and it has different causes due to the pregnancy trimester and the diagnosis and management would be crucial to prevent maternal-fetal life-threatening situations. In uncommon cases, varicose veins can emerge in the neck of the uterus, leading to a severe maternal hemorrhage.

**Case presentation:**

We presented a pregnant woman with vaginal bleeding and spotting during pregnancy with the diagnosis of cervical varix at 22 weeks of gestation. Close monitoring and proper patient education led to a term delivery at 37 weeks of gestation. Otherwise, an emergency postpartum hysterectomy after a cesarean section was performed due to uncontrolled bleeding from cervical varix.

**Clinical discussion:**

Although rare, cervical varix should be included in the differential diagnosis in a pregnant patient who appears with extensive vaginal bleeding to reduce maternal and/or neonatal morbidity or fatality. The approved diagnosis for that is not clear.

**Conclusion:**

This case report showed that Doppler and transvaginal sonography could be suitable diagnostic tools. The best management for cervical varix needs further research.

## Highlights

• Vaginal bleeding during pregnancy may lead to maternal-fetal life-threatening situations.
• In uncommon cases, varicose veins can emerge in the neck of the uterus, leading to a severe maternal hemorrhage.
• In significant vaginal bleeding, cervical varix should be considered as a possible cause.
• Doppler and transvaginal sonography could be suitable diagnostic tools.

## 1 Introduction

Varicose veins are abnormally enlarged veins commonly located on the lower extremities [1]. Pregnancy could be a risk factor for growing varicose veins [2]. Cervical varicose veins are rare among pregnant and only some cases have been reported [3,4]. Cervical varicose veins can cause life-threatening bleeding like other bleeding diseases such as placental abruption and placenta previa [[5], [6], [7]]. The diagnosis of cervical varicose veins was often confirmed after an episode of vaginal bleeding by color and power Doppler ultrasonography; however, the management of this condition was different in the reported cases including embolization, vaginal packing, and performing cerclage [4]. Since bleeding could be brutal for fetal-maternal health and the management and diagnosis of cervical varicose veins in pregnancy are not developed, we introduce a patient with cervical varices and the key roles of vaginal and Doppler ultrasounds and patient examinations. This case is written based on the SCARE checklist [5].

## 2 Case presentation

A 35-year-old pregnant woman gravida 2 para 1 (previous NVD (normal Vaginal Delivery)), at 22 weeks of gestational age, presented to an academic hospital with a history of fresh heavy, and painless vaginal bleeding. The patient was in stable but pale condition. Her vital signs were normal, but only her heart rate (HR) was slightly elevated (HR: 103/BP (blood pressure):100/70/RR (respiratory rate):16 /SO2:98 %). She did not complain of abdominal pain or uterine contractions. She only complained of occasional dizziness and headache but did not mention other poor signs such as nausea, vomiting, blurred vision, or diplopia. At the time of arrival, tocometry was reassuring (no pain was detected), and the performed speculum examination showed the bleeding at the spotting level.

The patient had a history of frequent vaginal bleeding that often occurred after defecation. Two occasions of these bleeding episodes had led to the reception of packed cells. The first time was at 14 weeks of gestational age with hemoglobin of 6.8 g/dL and the second time was at 18 weeks of gestational age with hemoglobin of 7 g/dL. On both occasions, the patient was discharged after receiving two packed cells with hemoglobin of about 9 g/dL and controlling bleeding. On another note, the patient's previous pregnancy ended without any problems at 40 weeks of gestational age.

**Fig. 1:**
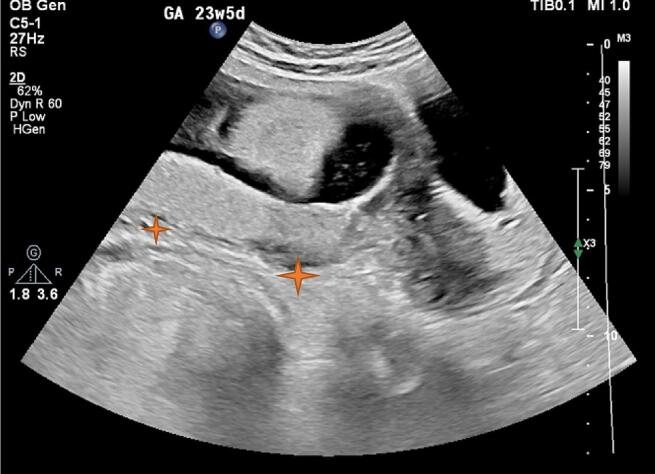
Abdominal sonography: The placenta was seen previa (stars), with no signs of hematoma behind it.

**Fig. 2:**
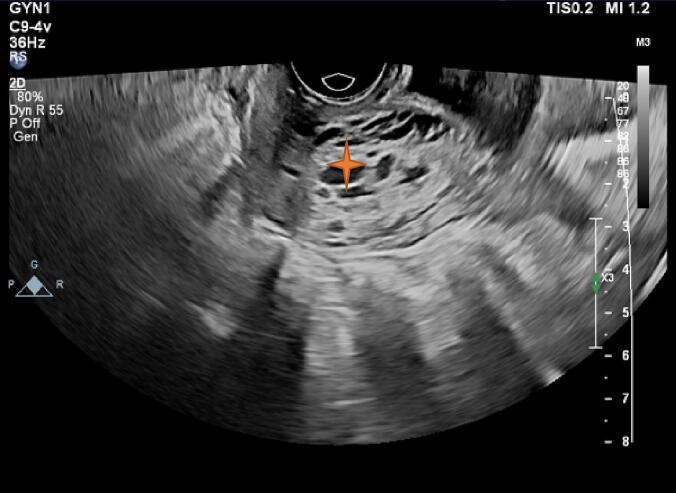
Transvaginal sonography, hypoechoic areas were suggestive of varicose veins, and fully active vessels were seen in the cervical region (star), especially in the anterior lip, which continued to the cervix.

**Fig. 3:**
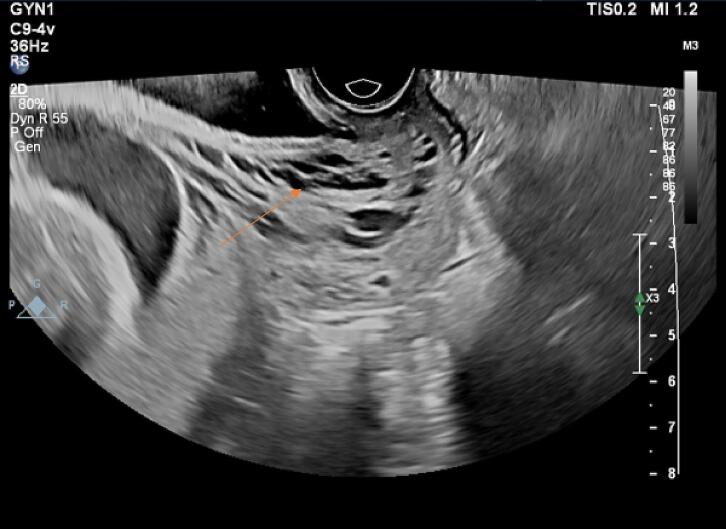
In the transverse-section of transvaginal sonography, blood vessels were fully extended to the circumference of the cervix (arrow).

**Fig. 4:**
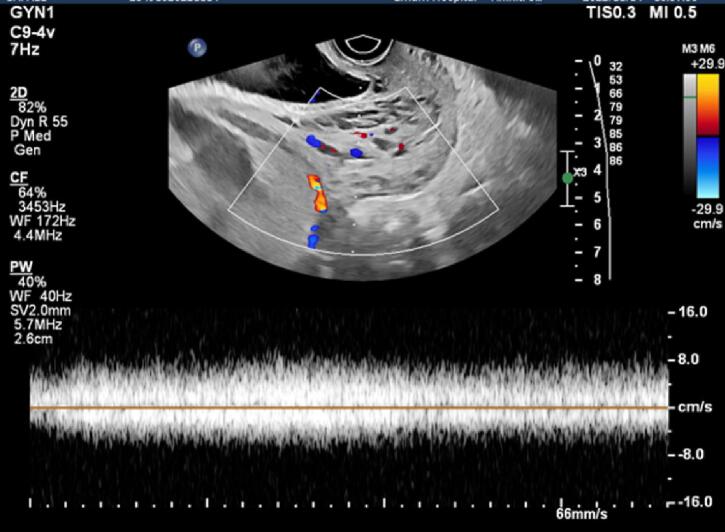
Color Doppler sonography showed venous flow in cervical varices.

In the ultrasound performed at the hospital, the placenta was previa, but no traces of hematoma behind the placenta were seen (Fig. 1). In the transvaginal sonography (TVS), hypoechoic areas were in favor of varicose veins, and fully active vessels were observed in the cervix region, especially in the anterior lip, which continued to the endocervix (Fig. 2). In the transverse section, the vessels were completely stretched to the circumference of the cervix (Fig. 3). Therefore, it was not possible to perform cerclage for the patient. Also, the color Doppler sonography showed us that the venous flow is flowing in the varicose veins of the cervix (Fig. 4). In addition, the myometrium line behind the placenta was clear, there were no abnormal vessels or lacuna in the placenta.

Finally, the patient was diagnosed with cervical varices according to both transvaginal and Doppler ultrasonography. She was observed and anti-constipation treatment was prescribed for her. Furthermore, the patient was prevented from having sexual intercourse and any heavy work, so that she did not experience any frequent bleeding. Due to the possibility of an emergency cesarean section (C/S), 12 mg of betamethasone and Magnesium Sulfate (4 g loading over 20 min and the maintenance dose of 1 g/h/12 h) were given at the appropriate time for the development of the fetal lung and neuroprotection. After that, she underwent an elective cesarean section at 37 weeks of gestational age due to the prevention of rupture of cervical varices at NVD. Also, the hysterectomy specimen was sent for histopathology and the result was dilated and tortuous cervical vessels without any findings suggesting a morbidly adherent placenta.

A female infant weighing 3400 g was delivered, with Apgar scores of 9 and 10 at 1 and 5 min. Extraordinary bleeding shortly after delivery happened from the varroosis of the cervix and the lower part of the uterus; the estimated blood loss was 2000 ml. The uterus was contracted and 1 g Tranexamic acid was injected intravenous, and immediately bilateral uterine artery occlusion and compression stitches in the lower uterine segment were done, although due to ongoing blood loss and failure to respond to conservative management. She underwent a total hysterectomy since the bleeding could not be controlled using compression stitches or medications to make the uterus contract. In addition, she received 4 units of packed cells, 2 units of FFP (Fresh frozen plasma), 2 units of PLT (platelets), 2 g of fibrinogen, and 2 g of Tranexamic acid during the cesarean section. The laboratory evaluation after the operation showed hemoglobin from 12.7 to 12.4 (g/dL). Also, Iron tablets were prescribed for her (200 mg/daily). She was discharged with no problem. At her follow-up visit one month after discharge, she appeared well, her anemia had resolved, and she continued exclusively breastfeeding.

## 3 Discussion

In this case report, we have faced a rare pregnancy condition that manifests with common symptoms. Also, we made the diagnosis for the patient according to her history of frequent vaginal bleeding and sonography (transvaginal and Doppler).

Due to some physiological changes during pregnancy, we might see more varicose veins compared to non-pregnancy. These changes include 1. dilation of venous vessels during pregnancy because of increased plasma volume 2. hormonal changes 3. increased intra-abdominal pressure 4. pressure from the pregnant uterus on the pelvic veins and inferior vena cava [[3], [4], [5], [6],^9^].

Although the cause of cervical varices in pregnancy is unknown, the lower placenta, placenta previa, and low-lying placenta could be some causes of this situation. The lower placenta increases blood flow to the cervix, which causes cervical veins to dilate and eventually cause cervical varices [8]. Moreover, exposure to diethylstilbestrol (DES) in mothers can cause pelvic vascular abnormalities in their daughters, which can be considered as one of the causes of this abnormality; also, female genital tract vascular malformations might have an association with cervical varices [7]. Furthermore, multiple gestations, IVF (in vitro fertilization), and maternal age of more than 35 could have an association with cervical varices among pregnant women [10].

The diagnosis of cervical varices could be made by physical examinations and ultrasonography (transabdominal or transvaginal), also MRI might be an aid if the diagnosis is not clear [11]. In this case, we used color and power Doppler sonography as paramedical diagnosis tools and hypoechoic areas showed the presence of varices in the cervix area.

Despite the rarity of this condition, the bleeding due to cervical varices can be life-threatening; therefore, suitable management is crucial for maternal-fetal health [12]. Literature reported some management for these patients including vaginal packing, blood transfusion, tocolysis, performing cerclage, emergency cesarean section, and uterine artery embolism; however, physical and pelvic relaxation was also recommended in most similar cases [7,11]. In this case, the patient was given physical and pelvic rest; also, it was not possible to perform cerclage due to the stretching of the varicose veins of the cervix. Moreover, we have performed an elective C/S for the patients to prevent any rupture due to varicose veins since only one case of NVD without problems has been recorded among these patients [7].

## 4 Conclusion

Although rare, cervical varix should be included in the differential diagnosis in a pregnant patient who appears with extensive vaginal bleeding to reduce maternal and/or neonatal morbidity or fatality. The approved diagnosis for that is not clear; however, this case report showed that Doppler and transvaginal sonography could be suitable diagnostic tools. The best management for cervical varix needs further research.

## Consent to participate and consent to publish

Written informed consent was obtained from the patient for the publication of this case report and accompanying images. A copy of the written consent is available for review by the Editor-in-Chief of this journal at the request.

## Ethical approval

The protocol of this report is approved by Tehran University of Medical Sciences, Tehran, Iran (reference code: 44.tums.733), 1 March 2023.

## Funding

Not applicable.

## Guarantor

Nafisseh Saedi, Marjan Ghaemi, Mona Moghadam, Mohammad Haddadi, Zeinab Hashemi, and Sedigheh Hantoushzadeh approved and accept full responsibility for the work and/or the conduct of the study.

## Research registration number

There is no register system in Iran for case report and we just register for ethical committee.

## CRediT authorship contribution statement

N.S. and S.H.: conceptualizing
F.G.V and Z.H. and S.S.: preparing data and pictures
M.M and M.G.: writing-original draft
M.H.: review and edit.

## Competing interest

The authors declare no competing interest.

## Data Availability

Data is available upon request.
